# Depression, Anxiety, Stress and Demographic Determinants of Hypertension Disease

**DOI:** 10.12669/pjms.306.5433

**Published:** 2014

**Authors:** Mamoona Mushtaq, Najma Najam

**Affiliations:** 1 Mamoona Mushtaq, PhD, Institute of Applied Psychology, University of the Punjab, Lahore, Pakistan.; 2 Najma Najam, PhD, Institute of Applied Psychology, University of the Punjab, Lahore, Pakistan.

**Keywords:** Anxiety, Depression, Stress

## Abstract

***Background and*** ***Objective*** ***:*** Research evidence supports the relationship of psychological and demographic factors with hypertension and these variables are strongest predictors of hypertension which are scarcely studied in Pakistan. The present study was carried out to explore the correlation of depression, anxiety, stress and demographic factors with hypertension.

***Method:*** We used correlation research design and a sample of (*N* = 237), hypertensive patients (*N* = 137) and their age matched healthy controls (*N *= 100) was taken from hospitals. Depression, Anxiety and Stress Scale (DASS) (Lovibond & Lovibond, 1995) was used to assess depression, anxiety and stress.

***Results:*** Results indicated significant positive correlation between depression (*χ*^2^_MH_ = 104.18, p < .001), anxiety (*χ*^2^_MH_ = 78.48, p < .001), stress (*χ*^2^_MH_ = 110.95, p < .001) and overall negative states (*χ*^2^_MH_ = 97.43, *p *< .001) with hypertension. Depression (*OR *= 1.44, p < .01), anxiety (*OR* = 1. 76*, p* < .01) stress (*OR* = 1.37, *p* < .01), job and dependents, working hours and weight turned out as predictors of hypertension.

***Conclusion:*** Hypertension has significant positive relationship with depression, anxiety, stress and with demographic variables. The findings of the present study will contribute in the existing knowledge of health professionals to enhance public awareness regarding the harmful outcomes of depression, anxiety and stress upon human health.

## INTRODUCTION

Escalating health problems in the world necessitate health professionals and researchers to investigate the factors responsible for the development of different diseases in human beings, and one of them includes hypertension. Pervasive increase of hypertension among different nations is a serious issue these days. There were 26% hypertensive adults in America in 2000.^1^ About one billion people are suffering from hypertension globally and the prevalence rate will increase up to 1.56 billion by 2025.^2^ It is reported that consistent high blood pressure is damaging the health of almost 25% of youngsters of both sexes.^3^ The alarming point is that more than 50% hypertensive patients do not even know that they are suffering from it.^4^

In Pakistan, the prevalence of hypertension is 34% in men and 24% in women.^5^ Hypertension is frequently prevalent in men after 35 years of age than women of that age. Additionally there are an estimated 12 million hypertensive patients in Pakistan.^6^ Furthermore, in Rawalpindi Division about 24.3% of the population over the age of 18 years and overall 36% of population is reported to have high blood pressure. Whereas reported 15% over the age of 18 years and 36% over the age of 45 years have the diagnosis of hypertension.^6^ Thus it appears to be rapidly increasing, neither treated nor controlled. Hence, it is emerging as a major health menace in the public health sector.^7^

Biological, social and psychological factors are often considered as significant risks of hypertension. Psychological state of an individual greatly affects the physical condition of human body. Empirical evidence reports high incidence of depression, anxiety and stress among patients with hypertension.^8^ Depression is widespread in hypertensive patients and relationship of depression with hypertension has been established by earlier researchers.^9^ In a study, individuals reporting high levels of hopelessness at baseline were found to be 3 times more likely to become hypertensive in near future.^10^ The research evidence also suggests that anxiety is another significant cause of increased blood pressure and is independent predictor of future hypertension.^11^^,^^12^

 Stress has been considered an important factor in the etiology of hypertension. Stress is known to be significantly correlated with hypertension and causes many cardiac problems.^13^ Natural reaction of the cardiovascular response to stress is the increase in heart rate. Young adults who have greater blood pressure response to stress may be at risk for hypertension as they are grown up.^14^

The role of demographic variables is vital in leading to hypertension. Marked social disparities in individual’s health exist across all nations of the world. Whether the indicator of socioeconomic status is education, income or occupational status, people belonging to low SES are at a greater risk of inducing sickness and easily become victims of disability or premature death than people belonging to high SES.^15^ Higher education affects health promoting behavior and resultantly causes lowering prevalence of overweight, which is an established risk factor of hypertension.^15^ Another important contributing variable to hypertension is overweight and obesity.^16^^,^^17^ Long and strenuous working hours which is the part and parcel of private job culture, is a significant risk factor of hypertension.^18^ Therefore the role of social variables need to be understood.^14^ In the present study, it was predicted that those who experience high level of depression, anxiety and stress, and with circumstantial difficulties consequently suffer from hypertension. In other words, are these factors correlated with hypertension?

Although the field of psychological risk factors of hypertension is not a new subject, still very few scientific studies have been carried out in developing countries especially in South Asia. Regardless of its significance, psychological aspects of hypertension have always been overlooked by researchers and physicians. Few researches conducted in this area are based upon the data drawn from lower masses only.^17^Growing literature on hypertension reports that hypertension control in Pakistan is partially achieved.^7^Therefore, the research was planned to explore the relationship of hypertension with depression, anxiety, stress, and demographic factors among hypertensive patients.

***Hypotheses:***

1. Depression, anxiety and stress would be positively correlated with hypertension among hypertensive patients.
2. Depression, anxiety, stress and other social variables would be significant predictors of hypertension.
3. There would be difference on depression, anxiety and stress between hypertensive men and women.

## METHODS

***Participants and procedure: ***We used co relational research design for this research. A sample of 237 participants, hypertensive men (*n* = 77), women (*n* = 60), non-hypertensive men (*n* = 50), and women (*n* = 50), was taken from outdoor departments of 2 public hospitals using a purposive sampling technique.

***Inclusion criteria:*** Inclusion criteria for hypertensive patients was (a) those patients who had currently been taking antihypertensive medicines (b) participants who were able to read and write Urdu language.

***Exclusion criteria:*** Patients suffering from chronic or terminal illness including (a) coronary heart disease (b) liver disease (c) renal disease (d) diabetes (e) malignant disease like cancer. 

***Non-hypertensive group***: They were matched to every case of hypertension for age (up to 3 years older and younger), gender, monthly income and working hours. Non-hypertensive group was taken from the hospital and they were the visitors or non-blood relatives of the cases diagnosed with hypertension, (b) participants with no past, current or family history of hypertension were included in the sample.

***Sample characteristics:*** The age range of the study participants was from 30 to 65 years (*M *= 43; *SD* = 8.24). The range of their number of dependents was from 0 to 11. Their weight ranged from 63 to 98 kg (*M *= 73; *SD* = 8.02), and working hours from 4 to 16 hours (*M *= 8.80; *SD *= 4.08).

**Table-I:** Demographic characteristic of the research participants (*N *= 237).

**Demographic variables**		**Hypertensives ** ** (** ***n*** ** = 137)**		**Controls** **(** ***n*** ** =100 ) **	
		***f***	***%***	***f***	***%***
Gender	Men	77	56	50	50
	Women	60	44	50	50
Occupation	No job	50	36	42	42
	Office job	62	45	36	36
	Business	17	13	18	18
	Both	8	6	4	4
Family history of hypertension	No	7	5	94	94
	Yes	130	95	6	6
Spouse job	No	88	64	53	53
	Yes	49	36	47	47

Official permission was obtained from hospital authorities for data collection from hypertensive patients and healthy controls who were visiting the hospital. Before administration of questionnaires participants were briefed about the purpose of study. A consent form, demographic information form and DASS were independently administered to all research participants. 

***Instruments:***

1. ***Demographic information questionnaire*** **:** Participants completed a comprehensive demographic information questionnaire which was prepared by the researchers regarding the age, marital status, education, occupation, monthly income, weight, number of children and dependents, family history of hypertension, spouse’s job and working hours of the research participants.
2. ***Depression, Anxiety and Stress Scale by Lovibond & Lovibond (1995)*** **:** ^19^ DASS is an internationally standardized protocol. It is a self report instrument designed to measure 3 relatively negative states of depression, anxiety and stress of an individual. It consists of 42 items. Each item has four optional responses which are scored on Likert scale from 0 (did not apply to me at all) to 3 (applied to me very much). Cronbach’s α = .91 for depression scale, 0.84 for anxiety scale and .90 for stress scale are reported by authors.^19^ In the present study standardized Urdu translation of DASS by Potangaroa (2006) was used.^20^

## RESULTS

***Relationship of depression, anxiety and stress with hypertension: ***Mentle Haenzel Chi-square test of linear association was applied for exploring relationship of depression, anxiety and stress with hypertension. If the exposure variable is ordinal, the ordinary chi-square test does not take into account the inherent order among the categories. It hardly checks the overall departure of observed from expected across the r ×2 cells of the table. A test of linear association (Pearson Chi-square) between columns and rows will be statistically insufficient, because it fails to distinguish between one and two category differences.^21^ In the present research each dimension of depression, anxiety and stress were categorized in to 3 levels like high, medium and low, but the levels are not given in the table because in all cases “high” was significantly related with hypertension.

**Table-II:** Relationship between depression, anxiety, stress and hypertension (*N =* 237)

**Variable **	***M***	***SD***	***α***	***χ*** ^2^_MH_ *** (df = 1)***
Depression	15.78	11.49	.91	104.18***
Anxiety	20.62	13.94	.84	78.48***
Stress	21.42	11.10	.90	110.95***
DASS	57.62	32.91	.91	97.43***

^Note:^^ M = Mean scores; SD = Standard deviation; ^^χ^^2^^MH ^^= Maentle Haenzel Chi-square^^; ^^α = reliability coefficient;^

*** ^= ^^*p <*^ ^.000.^

The results in the Table-II show that there is significant correlation of hypertension with depression, anxiety, stress and (DASS) (^***^p < .001). The reliability coefficients indicate that the scales were reliable for the present sample.

***Effect of depression, anxiety and stress on hypertension: ***Binary logistic regression model was run to find depression, anxiety and stress as predictors of hypertension. 

**Table-III:** Depression, anxiety and stress independently associated with hypertension in hypertensive cases and controls (*N *= 237).

**Variable **	*** B ***	***S.E***	***LL***	*** OR***	***UL***
Constant	-13.45**	4.90			
Depression	.36**	.13	1.10	1.44	1.88
Anxiety	.56^***^	.24	1.09	1.76	2.85
Stress	.31*	.15	1.01	1.37	1.85
DASS	.75^***^	.21	1.45	1.85	3.05

^ Note:^ ^R²^ ^= 55.51, Hosmer & Lemeshow), 270 (Cox & Snell), .68 (Negelkerke). Model χ2 (21) = 51.60; LL^

* ^*p <*^ ^.05,^

** ^*p <*^^. 01.^

***Analysis of coefficients: ***The odds ratio given in Table-III for depression is 1.44 and coefficient is positive. The value of the coefficient (.36) reveals that an increase of one unit scale in depression is associated with increase in the odds of hypertension development by a factor of 1.44 (95*%* CI, 1.10-1.88, *p < *.01). The odds ratio for anxiety is 1.76 and *B =* .56. The coefficient is positive and the odds ratio is 1.76, therefore as the anxiety increases by one scale unit, chances of hypertension in a person is increased 1.76 times. The *OR* for stress is 1.37 and coefficient is positive. The value of the coefficient (.45) reveals that an increase of one unit scale in stress is associated with increase in the odds of hypertension development by a factor of 1.37 (95*%* CI, 1.01-1.85, *p <* .001). Finally the value of combine effect of depression, anxiety and stress (DASS) come out as predictor of hypertension (95 *% *CI, 1.45-3.05*, p* < .001). The prediction value of R^2 ^= 55.51 indicates that model is adequately fit and psychological correlates are contributing 55.51*%* in the hypertension development. 

***Effect of social variables on hypertension:*** Logistic regression analysis was run to examine social variables as predictors of hypertension.

**Table-IV:** Demographic factors predicting hypertension (*N* = 237).

***Variable***	*** B***	***S.E***	** LL**	***OR***	** UL**
			** 95** ***%***	***CI***	
Constant	-4.13	.97			
Office job	.31**	.17	.91	1.14	1.72
Monthly income	-.45***	.18	1.74	1.23	1.97
Spouse’s job	-.42**	.23	1.05	1.64	1.83
Number of dependents	.34**	.15	.74	1.42	1.85
Weight	.28**	.12	.70	1.10	1.71
Working hours	.40**	.17	1.03	1.56	2.27

^ Note:^ ^R²^ ^= 62.43; Hosmer & Lemeshow); 27.31 (Cox & Snell), .71 (Negelkerke) Model χ2 (21) = 51.32,^

** ^*p <*^ ^.01,^

*** ^*p <*^ ^.001^

***Analysis of coefficients: ***The value of R^2 ^= 62.43 shows that model is adequately fit and social variables are contributing 62.43% in the hypertension. The odds ratio for office job is 1.14 and *B* = .31, and the coefficient is positive, therefore as office job increases by one scale unit chances of hypertension is increased 1.14 times. Protective effect of monthly income and spouse job is significant in hypertension. The odds ratio for monthly income is 1.23 and *B* = -.45. The coefficient is negative and odds ratio is 1.23, consequently as the income is increased by one scale unit chances of hypertension is decreased by a factor of 1.23 times. The odds ratio for spouse’s job is 1.64 and *B* = -.42, so as spouse job is increased by one scale unit chances of hypertension is decreased 1.64 times. The odds ratio for number of dependents is 1.42 and *B* = .34. The odds ratio is 1.42, each unit increase in the scores of number of dependents is associated with the odds of hypertension increase by a factor of 1.42 (95 *%* CL, .74-1.85). Similarly weight and working hours turned out as significant predictors of hypertension (95 *%* CL, .70-1.71, & 95 *%* CL, 1.03-2.27) respectively.

Difference of variables was also investigated and significant differences were observed between hypertensive men and women on depression (*M *= 19.82, *SD* 4.65 *& M =* 43.53, *SD = *10.58, *p <* .001), anxiety (*M =* 33.27, *SD =* 11.92 *& M =* 19.40, *SD* = 6.58, *p <* .001) and stress (*M =* 47.33, *SD *8.53 & *M =* 24.50, *SD =* 6.52, *p <* .001) respectively.

## DISCUSSION

The present research was conducted to explore the relationship of hypertension with psychological correlates and to find the significant predictors of hypertension. Inclusion of the control variables ensured that relationship between psychological variables and hypertension did not owe to these variables. The results of the current study indicate that hypertension has significant positive relationship with depression, anxiety, stress and with demographic variables. Furthermore depression, anxiety, stress, monthly income number of dependents, spouse’s job and working hours turned out to be significant predictors of hypertension.

The results reveal that there is significant relationship between depression and hypertension. This finding is in accordance with previous findings which concluded that depression is correlated with hypertension and also predicts hypertension.^18^ It is reported that the individuals experiencing high levels of hopelessness at baseline were 3 times more likely to become hypertensive in near future.^10^ However researchers also agree that depression and hypertension are reciprocally correlated, depression leads to hypertension^22^ and hypertension raises the level of depression.^12^

Additionally, as hypothesized relationship of hypertension with anxiety remained statistically significant and anxiety was observed a very serious disease which brings about harmful effects upon body.^22^ Enough research evidence supports anxiety as a single most cause of hypertension.^23^ It is also reported that participants developing hypertension at later stage, have significant anxiety at the baseline stage as compared to the participants who remained non hypertensive.^24^ Thus it may be concluded that anxiety and depression are significant predictors of hypertension.^10^

Moreover, stress has been considered a main cause in the etiology of hypertension.^13^In a study the significant effect of laboratory stress was greater upon hypertensives as compare to non-hypertensive controls.^13^ Existing literature has reported the relationship of depression, anxiety and stress with hypertension.^25^ Present findings are consistent with previous findings, which, convincingly demonstrate a positive correlation between psychological stress and hypertension.^6^^,^^13^^,^^15^ Hypertension may rightly be called an emotional disease. If the individual combats with severe conflict or frustration, uncertainty, impatience or deprivation; the result is stress. The successive stressful events play havoc in hypertension.^25^

Furthermore, job, monthly income, spouse’s job, number of dependents and weight turned out to be the significant social predictors of hypertension. A large number of hypertensive men appears to have workplace stress. Long working hours have emerged as a significant predictor of hypertension in the present research.^18^ In Pakistan, traditionally the family expenditures and finances are born by the men. Moreover, number of dependents appeared as a significant predictor of hypertension. This explains that more the number of family members more the expenditures would be. In Pakistan when women work to add family resources they protect their counterparts from being hypertensive as revealed in the current research. In the present research weight appeared as a significant predictor of hypertension, which is consistent with earlier researches in Pakistan.^16^^,^^17^ Hypertensive patients are not in the habit of going to gyms to work out and seldom or never do cardio exercises.

Finally significant gender differences were also seen between hypertensive men and women on depression, anxiety and stress. Thus the findings of current research establish the role of depression, anxiety, stress and social factors in developing hypertension. 

***Limitations: ***The present research was conducted with relatively small sample, thus the need for further replication is indicated. Moreover, we did not study the covariate factors such as BMI, type of food and smoking. Thus limiting the findings of present research.

***Implications: ***As reported by Jaffer, Chaturvedi, and Pappas, (2006) high prevalence rate of hypertension is found among children in Karachi city.^17^ The findings of the current research can be highlighted through media and public health awareness programs to prevent the future generations from hypertension. The early identification of negative emotions in causing hypertension in America has yielded some promising results in treating it. The findings of this research have implications for promoting the understanding of psychological and demographic factors of hypertension in Pakistani population.

## Author’s contribution:

**MM** conceived, designed, collected data, did statistical analysis & wrote manuscript and edited of manuscript.

**NN** review and final approval of manuscript.
